# Achieving Long‐Term Operational Stability of Perovskite Solar Cells with a Stabilized Efficiency Exceeding 20% after 1000 h

**DOI:** 10.1002/advs.201900528

**Published:** 2019-05-14

**Authors:** Tae‐Youl Yang, Nam Joong Jeon, Hee‐Won Shin, Seong Sik Shin, Young Yun Kim, Jangwon Seo

**Affiliations:** ^1^ Division of Advanced Materials Korea Research Institute of Chemical Technology (KRICT) 141 Gajeong‐Ro, Yuseong‐Gu Daejeon 34114 Republic of Korea; ^2^ Department of Energy Science Sungkyunkwan University Suwon 16419 Republic of Korea

**Keywords:** iodine migration, long‐term operational stability, oxygen, perovskite solar cells

## Abstract

Perovskite solar cells (PSCs) with mesoporous TiO_2_ (mp‐TiO_2_) as the electron transport material attain power conversion efficiencies (PCEs) above 22%; however, their poor long‐term stability is a critical issue that must be resolved for commercialization. Herein, it is demonstrated that the long‐term operational stability of mp‐TiO_2_ based PSCs with PCE over 20% is achieved by isolating devices from oxygen and humidity. This achievement attributes to systematic understanding of the critical role of oxygen in the degradation of PSCs. PSCs exhibit fast degradation under controlled oxygen atmosphere and illumination, which is accompanied by iodine migration into the hole transport material (HTM). A diffusion barrier at the HTM/perovskite interface or encapsulation on top of the devices improves the stability against oxygen under light soaking. Notably, a mp‐TiO_2_ based PSC with a solid encapsulation retains 20% efficiency after 1000 h of 1 sun (AM1.5G including UV) illumination in ambient air.

The power conversion efficiency (PCE) of perovskite solar cells (PSCs) has exceeded 22% with mesoporous TiO_2_ (mp‐TiO_2_) as the electron transport material (ETM) on a transparent conducting oxide.^1^ Despite such high PCEs, poor long‐term stability of mp‐TiO_2_ based PSCs is a problematic issue for commercialization. Photocatalytic chemical reaction with absorption of ultraviolet (UV) light by TiO_2_ is considered to induce degradation of PSCs.^2^ This effect was significantly weakened by simply using UV‐cut filters during light soaking tests in TiO_2_‐employed devices; however, the loss of power is inevitable as well. Introduction of luminescent photopolymers into PSCs for converting UV light to visible light is another solution, but is not cost effective.^3^ Imperfect interfaces, including charge recombination via deep traps at interfaces, are also known to induce the stability loss under illumination. Surface passivation of ETMs is considered as an effective method to prevent the degradation through this mechanism.^4^

Organic hole transport materials (HTMs) are also essential components for highly efficient mp‐TiO_2_ based PSCs but are a major cause of poor long‐term stability,^5^ while HTM‐free PSCs have showed excellent long‐term operational stability.^6^ Unlike Spiro‐OMeTAD, poly(triarylamine) (PTAA) combined with a quadruple‐cation perovskite has showed excellent long‐term thermal stability under illumination (without UV light) at inert nitrogen condition.^7^ Under similar condition, PSCs with an inorganic HTM without any additives, CuSCN, showed long‐term stability as well as a high PCE above 20%.^8^ Interestingly, although CuSCN‐incorporated PSCs were quite stable at high temperatures, long‐term stability under light soaking could not be achieved without a barrier layer between CuSCN and Au.

It should be also noted that atom/ion migration from metal electrodes or the organometallic halide perovskites into the HTM is one of the leading causes of the degradation.^9^ Recently, it is reported that the iodine migration in the perovskites is enhanced by illumination, which can be associated with the decomposition of the perovskite.^10^ Thus, suppressing the intrinsic degradation factors such as ion migration as well as overcoming the extrinsic degradation factors such as humid air and UV light is currently challenging to realize a long‐term stability of PCSs including mp‐TiO_2_ and organic HTMs under a practical operating condition.

We reason that operational stability of PSCs correlates with the external factors from the atmosphere because the stability of PSCs depends on encapsulations or the testing atmosphere.^11^, ^12^ In PSCs with wider bandgap oxides, such as SnO_2_, as ETMs, long‐term operational stability is not perfectly achieved.^13^ The long‐term operational stability of 1000 h in BaSnO_3_‐based PSCs was realized only in a laminate cell. Even small amounts of oxygen can affect performances of PSCs since oxygen can easily react with halides.^14^, ^15^ It has also been demonstrated that oxygen induces photodegradation of methylammonium lead iodide (MAPbI_3_).^16^, ^17^ Haque and co‐workers claimed that the degradation stems from the formation of reactive superoxide species in mediating halide vacancies in the perovskite materials.^16^, ^18^ In addition, organic semiconductors used as charge transport layers can also degrade when exposed to oxygen and light.^19^ Nevertheless, the effects of oxygen on the long‐term stability of PSCs have attracted only minor attention because it has been considered that the perovskites are much less susceptible to oxygen, rather than humidity and heat, even though oxygen is one of the most exposure species during the manufacture and the operation of PCSs. It is critical that the combination of oxygen and light as accelerated aging factors would cause the deterioration of the perovskites.

In this work, we investigate the effects of oxygen on the degradation of PSCs under illumination and reveal that charge transport from the perovskite to the HTM becomes ineffective by iodine migration before the degradation of bulk perovskite. Several papers have reported the degradation behavior of the devices and decomposition of perovskite materials by oxygen and ion migration; however, none of those have dealt with influences of oxygen on the operational stability of the devices. Most of them were carried out in ambient air including both moisture and oxygen; they could not specify the reason of the degradation and were more focused on the moisture rather than oxygen. Here, it is also demonstrated that mp‐TiO_2_ based PSCs have long‐term stability under illumination including UV if devices are immune to oxygen and humidity. PSCs exceeding initial PCEs of 20% have a device structure composed of fluorine doped tin oxide (FTO)/*d*‐TiO_2_/mp‐TiO_2_/(FAPbI_3_)_0.95_(MAPbI_3_)_0.05_/PTAA/Au.

When the devices are exposed to oxygen, PCE is reduced, and this degradation is accelerated by illumination. In order to clarify the effects of oxygen, the devices were illuminated in a gas‐tight purging chamber with O_2_ (purity >99.999%) flow as described in **Figure** **1**a. After devices were exposed to oxygen with 1 sun illumination, PCE reduced in half within an hour, (Figure 1b) which is much faster than the degradation in bulk properties such as absorption and photoluminescence of perovskite films in literature. While all photovoltaic parameters (*J*_sc_, *V*_oc_, and fill factor, FF) decreased with increasing the duration time of the O_2_–light exposure, FF was affected the most among the performance factors. The large decrease in FF indicates that transport of charge carriers becomes inefficient and/or the nonradiative recombination of photo‐excited carriers is enhanced. In addition, hysteresis in *J*–*V* curves increased because the drop of PCE for a forward voltage scan was larger than that for a reverse voltage scan. The broader hysteresis after the exposure is likely to be caused by more unbalanced charge transport between electrons and holes near perovskite/ETM or HTM interfaces.^20^ The performance of the degraded PSC was not recovered after aging the device in dark and in ambient air. Such degradation could be observed regardless of the types of perovskites, ETMs, and HTMs as well as device structure (Figures S1–S3, Supporting Information).

**Figure 1:**
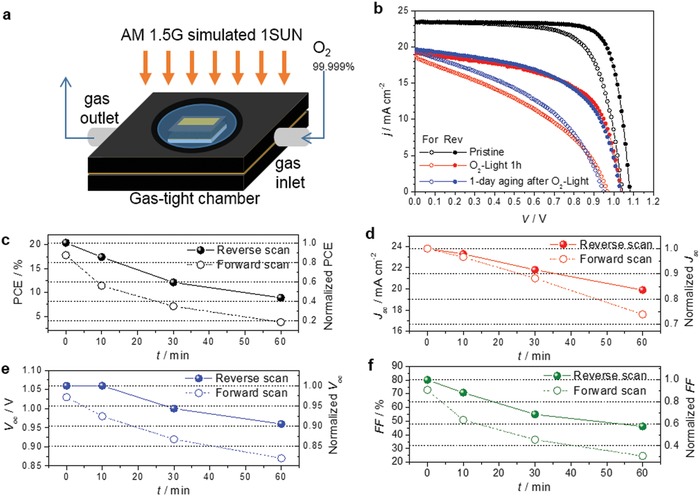
Variation of performances of perovskite solar cells (PSCs) with a device stack of FTO/*d*‐TiO_2_/mp‐TiO_2_/(FAPbI_3_)_0.95_(MAPbI_3_)_0.05_/poly(triarylamine) (PTAA) /Au. a) A schematic of the test system for the O_2_–light exposure. b) Current density versus voltage (*J*–*V*) plots under 1 sun illumination of pristine devices (black), and devices after 1 h of light soaking in O_2_ (red), and after 1 day of aging under dark and ambient air conditions (blue). Plots of c) PCE, d) short‐circuit current density (*J*_SC_), e) open‐circuit voltage (*V*_OC_), and f) fill‐factor (FF) as a function of the duration of the O_2_–light exposure.

On the other hand, when PSC devices were exposed to Ar atmosphere under illumination or to O_2_ atmosphere in the dark for an hour, a little degradation in *V*_oc_ and FF was observed, but their performances were completely recovered after the 1 day aging in the dark (Figure S4, Supporting Information). However, even under dark conditions, the higher pressure of O_2_ induced the permanent degradation of PCSs (Figure S5, Supporting Information). Consequently, oxygen ingress into PSCs is a main cause of the device degradation, and illumination seems to accelerate the degradation in the presence of O_2_. Considering the results of Ar‐light exposure, we can also exclude photocatalytic effect of TiO_2_ with UV light as one of the many causes of the photo‐induced degradation of the devices.

In order to elucidate the cause of the degradation of PSCs by oxygen, we investigated the change in time‐resolved photoluminescence (TRPL) of the perovskite thin film with and without charge transport layers according to the O_2_ exposure (**Figure** **2**). When a perovskite single layer and a mp‐TiO_2_/perovskite bilayer were exposed to O_2_ and light, photoluminescence (PL) decay curves were slightly extended (Figure 2a). The PL enhancement in the perovskite under illumination in O_2_ atmosphere is explained by the curing of trapping sites as claimed in literature.^15^ The transient behavior in the short‐time range (<100 ns) for the mp‐TiO_2_/perovskite double layers, which involves with the electron transport from the perovskite to the TiO_2_, did not change much by the O_2_–light exposure (Figure 2b). However, the PL decay curves for the perovskite/PTAA bilayer were largely extended after the O_2_–light exposure. (Figure 2c) After O_2_ exposure under dark, only the perovskite/PTAA sample showed slightly extended PL decay. These results indicate that the degradation of PSC devices by oxygen stems from the interruption of the transport of photo‐excited holes from the perovskite into PTAA.

**Figure 2:**
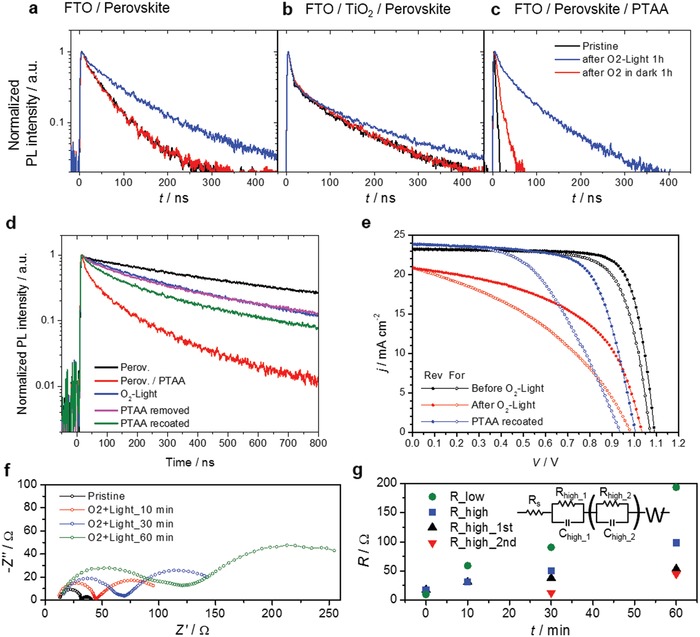
Time‐resolved photoluminescence (TRPL) decay curves of a) the perovskite film, b) perovskite film on TiO_2_ (*d*‐TiO_2_/mp‐TiO_2_), and c) perovskite film covered with PTAA. All samples are coated on FTO‐coated glass substrates. d) TRPL decay curves of the perovskite (black) and PTAA‐coated perovskite (red) on FTO substrates, FTO/perovskite/PTAA stacked samples applied with the following consecutive sequences: after 1 h of O_2_–light exposure (blue), then after removal of the PTAA layer (perovskite exposed to air again, magenta), and then after coating a new PTAA layer (green). e) *J*–*V* curves for a device before (black) and after 1 h of the O_2_–light exposure (red), and after coating a new PTAA and Au on the device in which the degraded PTAA was removed (blue). f) Nyquist plots at *V*_OC_ and g) electrical resistance corresponding to semicircles in Nyquist plots as a function of the duration of the O_2_–light exposure. Inset: An equivalent circuit for plotting the Nyquist plots (R_low represents a Warburg impedance).

The hole transport can be hindered most probably due to the barrier formation at the interface between the perovskite and the HTM or the degradation of the HTM from reducing electrical conductivity. We designed experiments to reveal where the hole extraction is hindered: 1) only the PTAA layer in the degraded device was washed out by using toluene, and 2) then, a new PTAA layer was recoated via spin‐coating on top of the perovskite film in the degraded device and a new Au electrode was deposited on top. TRPL measurements and *J–V* measurements were conducted before and after the O_2_–light exposure and the removal and recoating of the PTAA layer (Figure 2d,e).

Interestingly, the device in which the PTAA layer was removed showed a similar PL decay curve to the degraded device by the O_2_–light exposure. It is likely that the PTAA in the degraded device cannot extracts holes. When a new PTAA layer was coated on the sample, the PL decay curve was shortened but was still much longer than that of the pristine sample (denoted by Perov./PTAA). Replacing the PTAA layer restored the performance of the PSC partly, but it was still not perfect as well. The PCE of the devices rose from 11.0% to 16.2% for reverse scans by replacing the degraded PTAA with a new one but was lower than the initial PCE of 20.3%. While *J*_sc_ was fully recovered, FF increased from 51% to 67%; neither *V*_oc_ nor hysteresis was improved. Removing the PTAA and coating a new PTAA layer on a pristine sample have little effect on the performance of the device (Figure S6, Supporting Information). The incomplete recovery of the PL decay curves and *J–V* curves illustrate that the O_2_–light exposure induces degradation of the perovskite near the interface with the PTAA as well as degradation of the PTAA layer.

The interface between the PTAA and the Au electrodes does not contribute to the degradation by the O_2_–light exposure. When Au electrodes on the degraded device were replaced by newly deposited Au electrodes after removing old ones, the performance of the device was not recovered (Figure S7, Supporting Information).

We also observed that interfacial adhesion between the perovskite and the PTAA strengthened by the O_2_–light exposure in this experiment. In order to remove Au electrodes from devices, a tape detaching method was employed. The PTAA layer on the degraded device was not completely removed by detaching tape, while the PTAA layer in pristine device was removed clearly (Figure S8, Supporting Information). This result can be an evidence on chemical interaction between the perovskite and the PTAA.

The hindered hole transport leads to increase of series resistance of the devices. The increase of series resistance can be directly measured by analyzing impedance spectra (Figure 2f,g). Pristine PSC devices show two semicircles in Nyquist plots. The first semicircle at high frequencies corresponding to series resistance of the devices was significantly enlarged with increasing the duration of the O_2_–light exposure. Such a similar observation was reported in a previous work where the device with MAPbI_3_ and Spiro‐OMeTAD was exposed to light under ambient air condition.^12^ Even though a humid air atmosphere, this also supported our finding that the O_2_–light exposure could cause a deterioration of the perovskite/HTM interface of the device.

It should be noted that the optical absorbance, surface morphology, and crystallinity of the perovskite film were not varied after the O_2_–light exposure for 2 h. (Figures S9 and S10, Supporting Information). In addition, absorbance and conductance of PTAA on a thin layer of the perovskite do not change by the O_2_–light exposure (Figure S11, Supporting Information). These results indicate that rather than the bulk properties of the perovskite and PTAA, the interfacial properties are more likely to be affected by the exposure.

Next, depth profiles of time‐of‐flight secondary ion mass spectrometry (ToF‐SIMS) for the degraded PSC devices were compared to that of a pristine device (**Figure** **3**a,b). In the degraded devices, interestingly, we observed no change in the oxygen profile, whereas we found a large variation in the iodine profile. Iodine concentration in the PTAA layer was increased after the O_2_–light exposure. The accumulation of iodine at the Au/PTAA interface could also be identified. Larger amounts of iodine within PTAA and the interface were observed in devices with longer duration of the O_2_–light exposure (Figure 3c).

**Figure 3:**
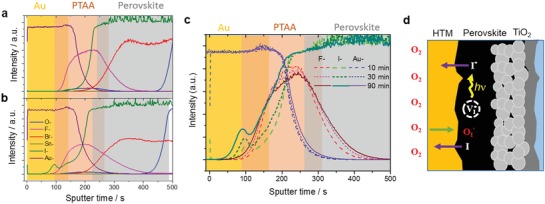
Depth profile of elements in PSCs from time‐of‐flight secondary ion mass spectrometry (ToF‐SIMS) measurements a) before and b) after 1 h of O_2_–light exposure. c) Variation of depth profiles with increasing exposure duration. Diffusion of iodine ions within the PTAA especially increases with longer exposure times to O_2_ and light. d) Schematic to describe the activation of iodine diffusion by oxygen and light.

The incorporation of iodine into PTAA seems to inhibit the hole transport. The existence of iodine in PTAA stems from the diffusion of iodine from the perovskite to the PTAA layer by O_2_ and light. Photo‐excited holes bind with iodine ions, thus forming neutral iodine.^10^ The photo‐excited neutral iodine with high activity might tend to diffuse into PTAA in which iodine activity is much lower. In addition, several experimental results show that O_2_ provides enhanced iodine activity and creates iodine vacancies in the perovskite,^21^ thereby activating the diffusion of iodine out of the perovskite. The diffusion of iodine into PTAA was also observed in the degraded device by exposing to high‐pressure O_2_ in the dark. If iodine is irreversibly removed by a condensed matter sink such as the HTM, the perovskite is eventually degraded into a nonstoichiometric phase.

To address the solution to prevent photo‐induced degradation in oxygen‐contained atmosphere, we can consider two approaches: 1) suppression of the diffusion of iodine and 2) isolation of devices from oxygen. For the first solution, a np‐Al_2_O_3_ layer was inserted between the perovskite and PTAA as a diffusion barrier. This device showed good stability against the O_2_–light exposure (**Figure** **4**a). The PCE of the devices was slightly decreased just after the exposure, but it was recovered after aging for a day under dark in ambient air conditions. The suppression of the iodine diffusion from the perovskite into PTAA layer was apparently confirmed by ToF‐SIMS depth profiling (Figure S12, Supporting Information). When the np‐Al_2_O_3_ layer was inserted not between the perovskite and PTAA but between PTAA and Au, it could not inhibit the degradation induced by the O_2_–light exposure (Figure S13, Supporting Information). These findings imply that the np‐Al_2_O_3_ layer is not enough as a barrier layer to prevent the outside oxygen from penetrating into the device but is still effective to suppress iodine diffusion from the perovskite to the PTAA layer. The improved long‐term stability of PSCs with adding of a np‐Al_2_O_3_ buffer in a literature seems to attribute to this effect.^22^

**Figure 4:**
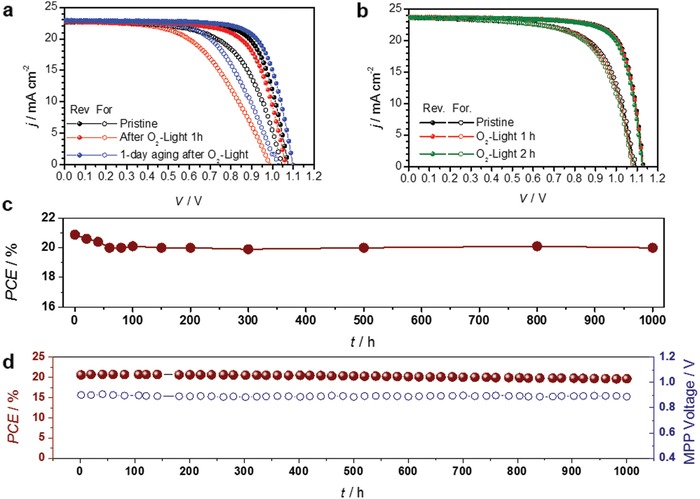
Suppression of the degradation induced from exposure to O_2_ and long‐term device stability of encapsulated perovskite solar cells. *J–V* scans of devices a) with a np‐Al_2_O_3_ layer between the perovskite and PTAA layers and b) with encapsulation. c) Shelf test results of the encapsulated device under 85 °C and 85% RH conditions and d) long‐term operational stability of the encapsulated devices under simulated real operating conditions (maximum power point tracking under 1 sun AM1.5G illumination including UV without controlling temperature in ambient air).

For the second solution, we have encapsulated PSC devices by laminating face‐sealing adhesive sheets on the top of the devices. In order to remove trapped air during the lamination and to improve the adhesion between the devices and the adhesive sheets, the encapsulated devices were aged in an autoclave. Then, the devices were completely protected against the ingress of oxygen and moisture. As shown in Figure 4b, the device is not degraded at all after 2 h of the O_2_–light exposure.

We also examined the long‐term stability of the encapsulated PSCs. First, we conducted a shelf test under 85 °C and 85% relative humidity (RH) conditions to evaluate encapsulation efficiency (Figure 4c). PCE of the device was maintained up to 96% of its initial PCE after 1000 h (from 20.9% to 20.0%). In this case, only 0.9% efficiency decrease was found in early burn‐in stage due to slight decrease of *J*_SC_ and FF. There is no additional drop in the device performance. This assures that the encapsulation of the device is fairly solid.

The long‐term operational stability under real operating conditions was also investigated with the encapsulated device (Figure 4d). Surprisingly, the PCE retained 96% of its initial value (20.6%) after 1000 h operation (maximum power point tracking, MPPT) under 1 sun AM1.5G illumination of a xenon lamp including UV despite using mp‐TiO_2_ as the ETM. The final PCE from a *J*−*V* sweep was 20.0%. Even though PSCs were encapsulated with the face‐sealing adhesive sheets, the long‐term stability of the PSCs was not guaranteed without the aging in the autoclave. As a result, a successful long‐term stability of PSCs can be realized by a solid encapsulation to completely block oxygen and even moisture in the atmosphere, thereby impeding the ion movement as well as the sublimation of volatile components from the perovskite.

Overall, the effects of oxygen on the operational stability of PSCs were systemically investigated in this study. PSCs under illumination quickly degraded in oxygen atmosphere due to hindering charge transport from perovskite to HTMs. We revealed that the degradation is induced by iodine diffusion into the PTAA layer without applying electrical bias or heat. Diffusion of iodine from the perovskite into the PTAA layer induces reduced hole transport ability near the interface between the perovskite and the PTAA. Pure oxygen atmosphere is an accelerated condition as compared with ambient atmosphere; however, it seems that oxygen has critical role on the photo‐induced degradation of PSCs. Long‐term stability of mp‐TiO_2_ applied PSCs can be simply attained by protecting the devices from oxygen and moisture, since even a small amount of oxygen notably affects the photo‐induced degradation of PSCs for a long‐term period.

## Experimental Section

*Device Fabrication*: A 60 nm thick dense blocking layer of TiO_2_ (bl‐TiO_2_) was deposited onto an FTO (Pilkington, TEC8) substrate by spray pyrolysis deposition carried out using a 20 × 10^−3^ m titanium diisopropoxide bis(acetylacetonate) solution (Aldrich) at 450 °C. 100 nm thick mesoporous TiO_2_ (TiO_2_ nanoparticles: average particle size = ≈50 nm, crystalline phase = anatase) films were spin‐coated onto the bl‐TiO_2_/FTO substrate using diluted pastes (SC‐HT040, ShareChem, Korea) and were calcined at 500 °C for 1 h in air to remove the organic portion. To fabricate perovskite solar cells based on (FAPbI_3_)_0.95_(MAPbBr_3_)_0.05_, the perovskite solution was prepared by dissolving 889 mg mL^−1^ of FAPbI_3_, 33 mg mL^−1^ of MAPbBr_3_, and 33 mg mL^−1^ of MACl in DMF/DMSO (8:1 v/v) mixed solvent. Then, the solution was coated onto the mp‐TiO_2_/bl‐TiO_2_/FTO substrate by two consecutive spin‐coating steps, at 1000 and 5000 rpm for 5 and 20 s, respectively. During the second spin‐coating (5000 rpm) step, 1 mL of ethyl ether was poured onto the substrate after 15 s. The intermediate phase substrate was then put on a hot plate at 150 °C for 10 min. After that, PTAA were spin‐coated as hole transport materials. PTAA solution in toluene (11 mg mL^−1^) was prepared, and 3.7 µL Li‐bis(trifluoromethanesulfonyl) imide (Li‐TFSI) in acetonitrile (340 mg mL^−1^) and 3.7 µL 4‐*tert*‐butylpyridine (*t*BP) were added. The PTAA solution was spin‐coated on (FAPbI_3_)_0.95_(MAPbBr_3_)_0.05_/mp‐TiO_2_/*d*‐TiO_2_/FTO substrate at 3000 rpm for 30 s. These all processes were carried out in air with controlling temperature (≈23 °C) and humidity (≈20% RH). Finally, the 70 nm thick Au counter electrode was deposited by thermal evaporation. The active area of this electrode was fixed at 0.16 cm^2^.

*Characterizations and Measurements*: The morphologies of the perovskite film coated with or without PTAA and the prepared devices were investigated using field emission scanning electron microsope (FE‐SEM, Tescan Mira 3 LMU FEG).

The absorption spectra were obtained using a UV–vis spectrophotometer (Shimadzu UV 2550) in the wavelength range of 300 to 900 nm.

The photovoltaic properties of the devices were measured using a solar simulator (Oriel Class A, 91195 A, Newport) with a source meter (Keithley 2420) at AM1.5G 100 mA cm^−2^ of illumination and a calibrated Si‐reference cell certificated by National Renewable Energy Laboratory (NREL). The *J*−*V* curves were measured along reverse scan direction from 1.3 to −0.2 V or forward scan direction from −0.2 to 1.3 V. The step voltage and dwell time were fixed at 10 mV and 50 ms, respectively. The *J*–V curves of all the devices were measured in ambient air (25 °C, ≈30% RH) by masking the active area using a metal mask with an area of 0.096 cm^2^.

Time‐resolved photoluminescence decay profiles were measured at 780 nm with 550 nm excitation of optical parameter oscillator laser system (NT 342A‐10‐AW, EKSPLA). The pulse energy was strongly attenuated to less than 1 µJ, in order to avoid nonlinear effects such as exciton–exciton annihilation. Emissions from samples were collected by a monochromator (SP2150, Princeton Instruments) equipped with a photomultiplier tube (PMT, Hamamatsu H10721‐20). The output signal from a PMT was recorded with a 500 MHz digital oscilloscope (DSO‐X 3054A, Agilent).

Depth profiles of elements were measured by time‐of‐flight secondary ion mass spectrometer (ION‐TOF GmbH) equipped with a 1 keV Cs^+^ primary ion beam for the sputtering and 30 keV Bi^+^ pulsed primary ion beam for the analysis.

Impedance spectroscopy measurements were performed by using an electrochemical impedance spectroscopy (model PGSTAT302N, AUTOLAB). The AC voltage was 0.2 V, and the frequency was swept from 1 MHz to 10 mHz. The values of resistance and capacitance were determined by fitting the Nyquist plots of the impedance (Z′′ vs Z′) using the software ZView (Version 3.1c, Scribner Associates Inc.).

The X‐ray diffraction (XRD) spectra were measured using a Rigaku Smart Lab X‐ray diffractometer to identify the crystal phase of the prepared films.

*Long‐Term Stability Tests*: For long‐term stability tests, PSC devices were encapsulated by laminating face‐sealing adhesive sheets developed for organic electronic devices.^23^ The lamination was conducted at 60 °C of roll temperature in dry air.

Damp‐heat shelf test was carried out in the chamber in which temperature and relative humidity were maintained at 85 °C and 85% RH, respectively. PCE of the device was periodically measured under AM1.5G simulated sun light after cooling the device down to room temperature.

Long‐term operational stability tests were performed by maximum power point tracking using a source meter (Keithley 2420, USA) operated by the self‐written software with a perturb–observe algorithm. A 450 W xenon lamp (Oriel, USA) with an AM1.5G filter was used as a light source, and light intensity was calibrated by a silicon reference cell. The temperature of the devices under the light soaking was not controlled. Ambient temperature and humidity were maintained at 25 °C and 30% RH, respectively.

## Conflict of Interest

The authors declare no conflict of interest.

## Supporting information

Supplementary
