# Supplementary material for: Achieving Long‐Term Operational Stability of Perovskite Solar Cells with a Stabilized Efficiency Exceeding 20% after 1000 h
Source: Adv Sci (Weinh). 2019 May 14;6(14):1900528. doi: 10.1002/advs.201900528 (PMC6661943; doi:10.1002/advs.201900528)
Supplement: Supplementary file 1 — Supplementary [file ADVS-6-1900528-s001.pdf]

Copyright WILEY-VCH Verlag GmbH & Co. KGaA, 69469 Weinheim, Germany, 2019.

Supporting Information

for *Adv. Sci.,* DOI: 10.1002/advs.201900528

Achieving Long-Term Operational Stability of Perovskite Solar Cells with a Stabilized Efficiency Exceeding 20% after 1000 h

*Tae-Youl Yang, Nam Joong Jeon, Hee-Won Shin, Seong Sik Shin, Young Yun Kim, and Jangwon Seo**

WILEY-VCH

Copyright WILEY-VCH Verlag GmbH & Co. KGaA, 69469 Weinheim, Germany, 2019.

Supporting Information

Achieving Long-term Operational Stability of Perovskite Solar Cells with a Stabilized Efficiency Exceeding 20 % after 1,000 Hours

*Tae-Youl Yang,[a †], Nam Joong Jeon,[a †] Hee-Won Shin,[b] Seong Sik Shin,[a] Young Yun Kim,[a] and Jangwon Seo[a] **

Changes in characteristics of PSCs after the O_2-light exposure

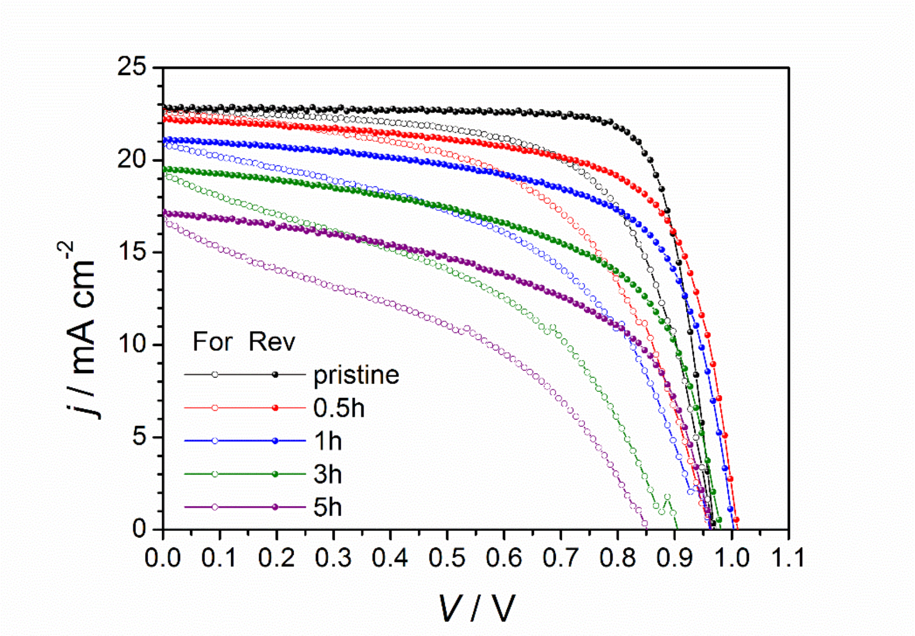

Figure S1. *j*-*V* plots for a $(FAPbI_3)_{0.95}(MAPbBr_3)_{0.05}$ / Spiro-OMeATD pristine device (black) and after the O_2-light exposure for 0.5 (red), 1 (blue), 3 (green), and 5 h (purple).

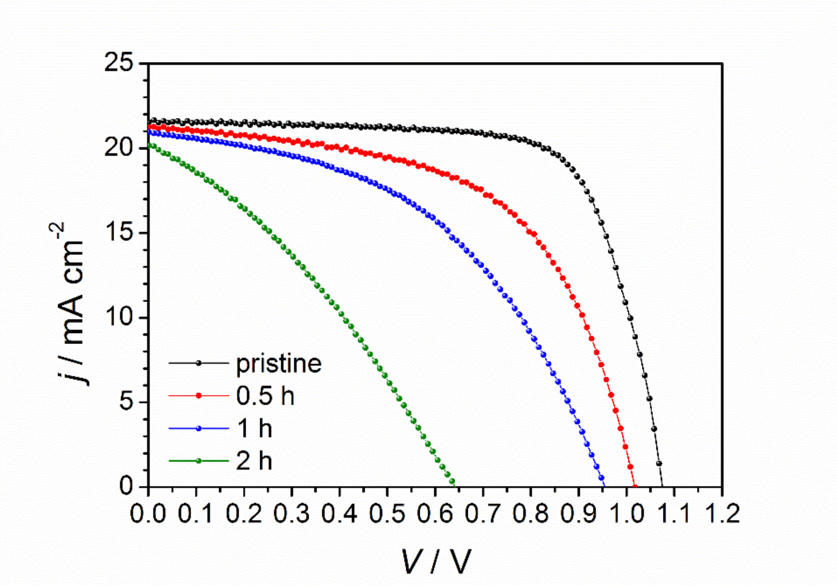

Figure S2. *j*-*V* plots for an $MAPbI_3$ / PTAA pristine device (black) and after the O_2-light exposure for 0.5 (red), 1 (blue), and 2 h (green).

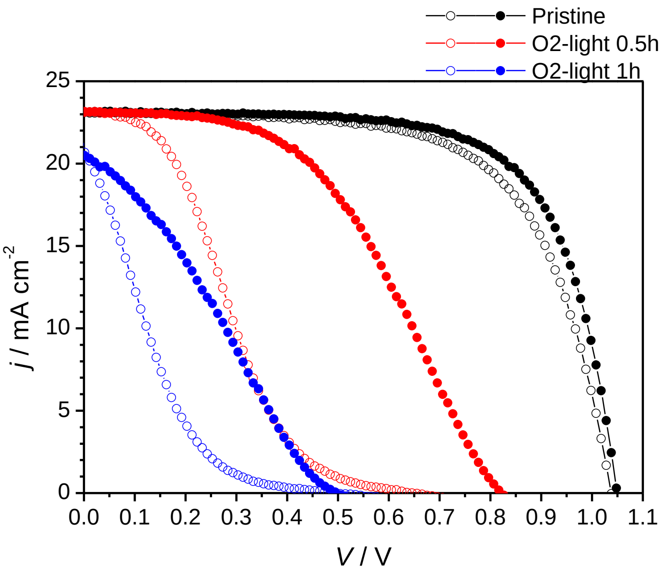

Figure S3. . *j*-*V* plots for a device with the planar structure ITO / SnO_2 / $(FAPbI_3)_{0.95}(MAPbBr_3)_{0.05}$ / PTAA / Au before and after the O_2-light exposure for 0.5 (red) and 1 hour (blue)

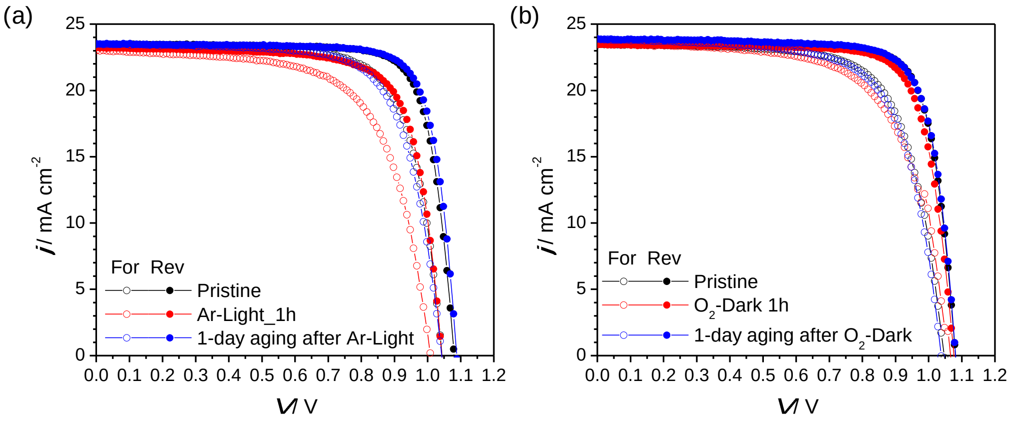

Figure S4. Current density vs. voltage (*j*-*V*) plots under 1-sun illumination of pristine devices (black), and devices after 1 h of light soaking (a) in Ar and (b) after 1 h of O_2 exposure in the dark (red), and after 1 day of aging under dark and ambient air conditions (blue).

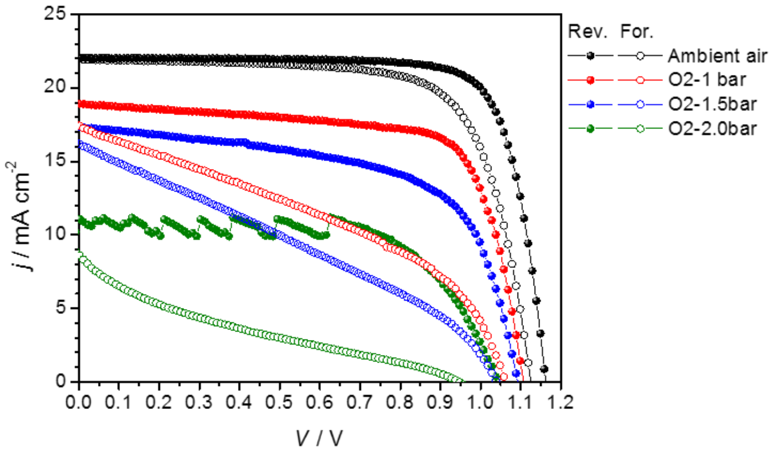

Figure S5. Degradation of perovskite solar cells (PSCs) by exposing to pressurized oxygen atmosphere for 1 h in the dark with various oxygen pressures.

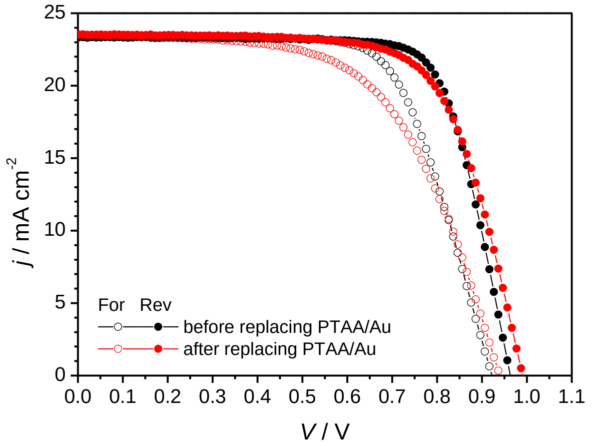

Figure S6. *j*-*V* curves of a pristine device before and after erasing the existing PTAA/Au and the re-deposition of PTAA/Au without O_2-light exposure.

Comparison of PSCs with and without the O_2-light exposure

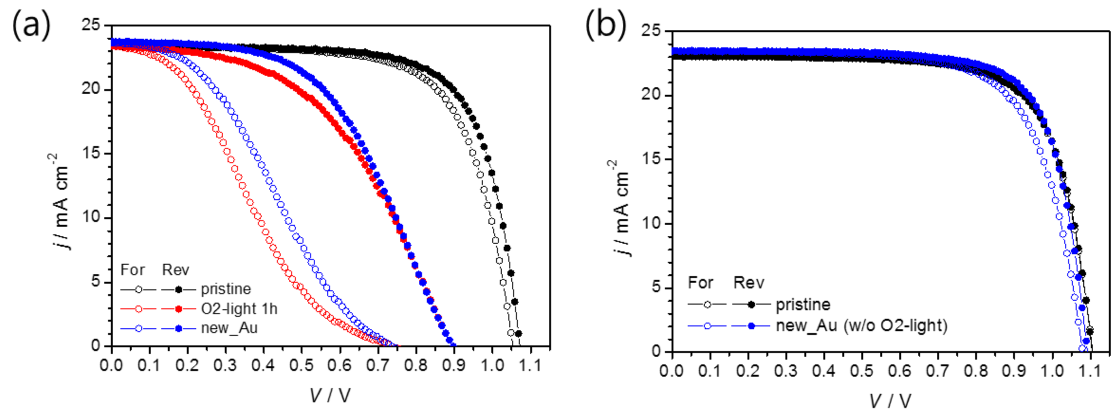

Figure S7. (a) j-V curves for a device before (black) and after 1h of the O2-light exposure (red), and after replacing Au electrodes with newly deposited ones on the degraded device (blue). (b) j-V curves for a pristine device before (black) and after replacing of Au electrodes with newly deposited ones (blue).

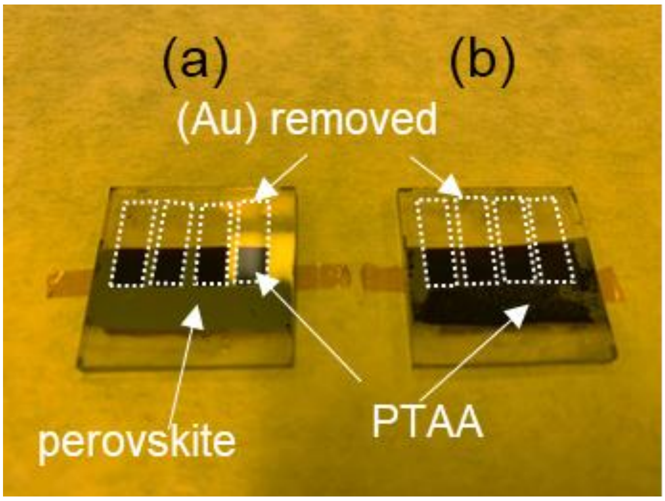

Figure S8. Images of devices in which PTAA and Au layers were removed with detaching Scotch® Magic™ tape from the top surface.

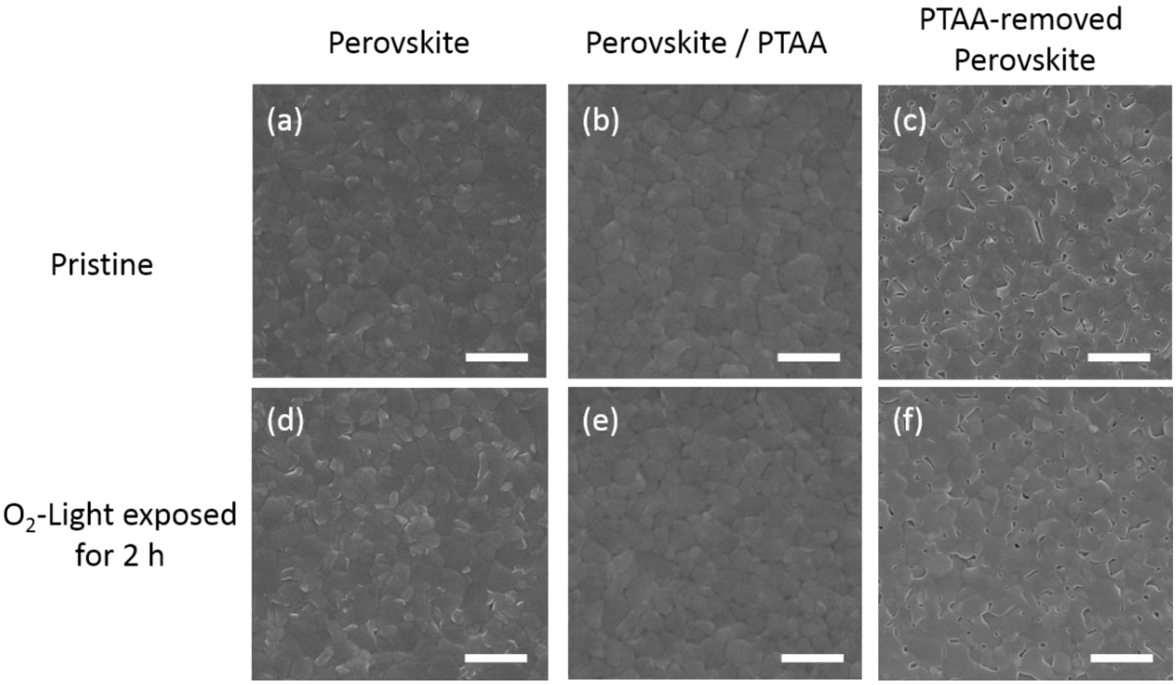

Figure S9. Comparison of surface morphologies before and after the O_2-light exposure of the sample of (a, d) a $(FAPbI_3)_{0.95}(MAPbBr_3)_{0.05}$ film, (b, e) a PTAA-coated $(FAPbI_3)_{0.95}(MAPbBr_3)_{0.05}$, and (c, f) a $(FAPbI_3)_{0.95}(MAPbBr_3)_{0.05}$ after removal of PTAA. Scale bars indicate 2 μm.

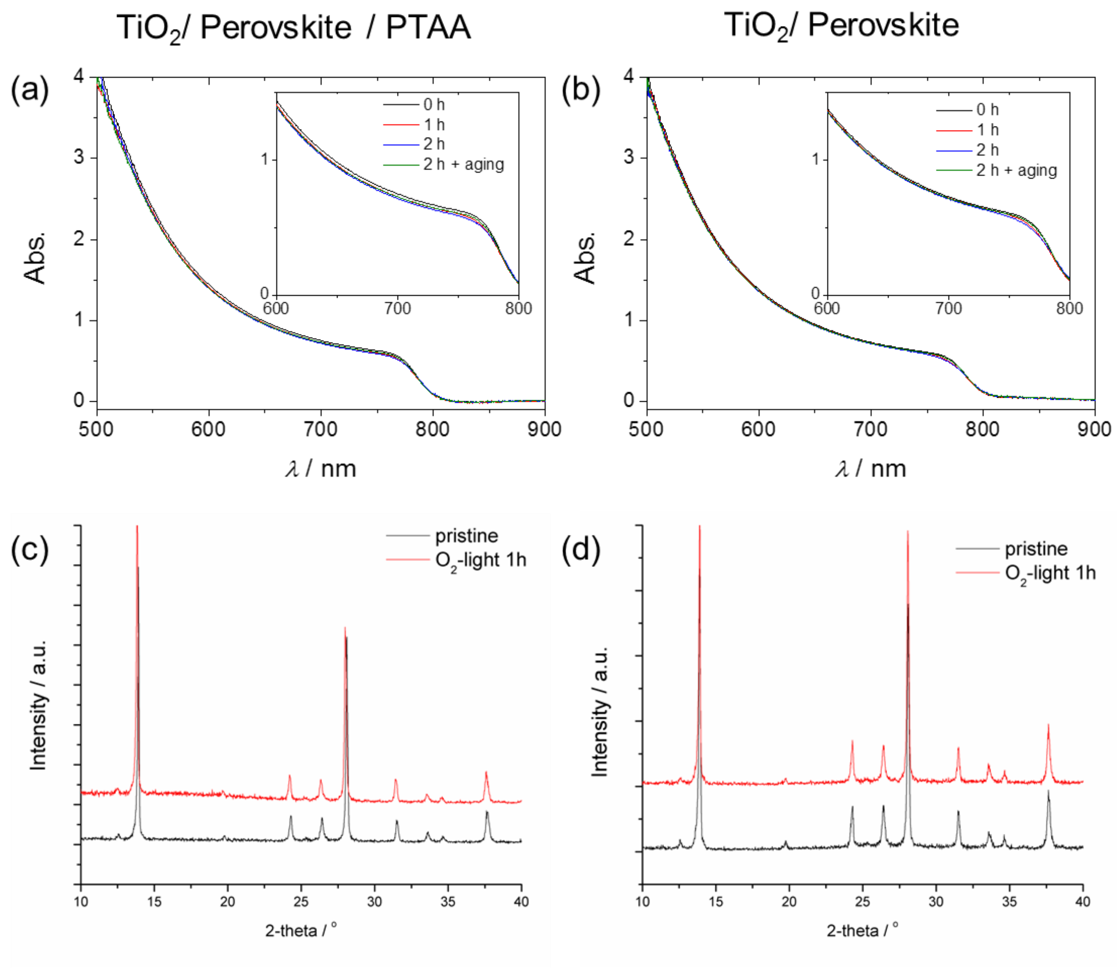

Figure S10. UV-Vis and X-ray diffraction (XRD) spectra for devices with stacks of (a, c) FTO / TiO_2 / $(FAPbI_3)_{0.95}(MAPbBr_3)_{0.05}$ / PTAA and (b, d) FTO / TiO_2 / $(FAPbI_3)_{0.95}(MAPbBr_3)_{0.05}$ before and after the O_2-light exposure.

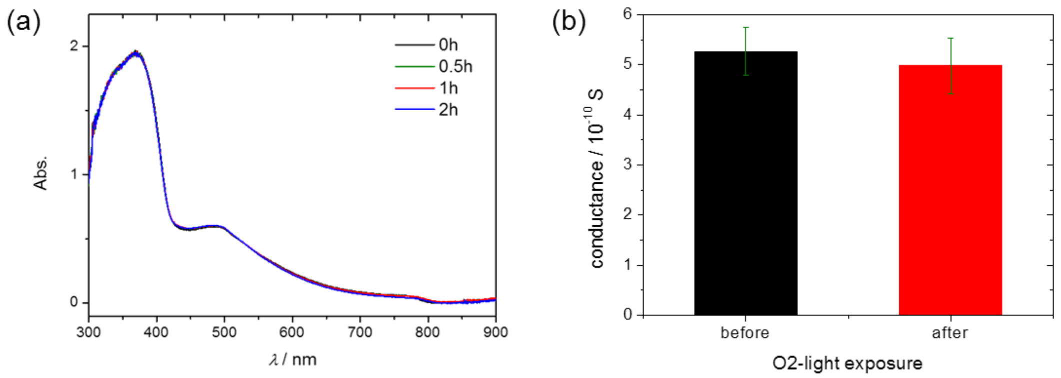

Figure S11. (a) UV-Vis absorption spectra for a PTAA / thin perovskite bilayer sample with increasing the duration of the O_2-light exposure. (b) The out-of-plane conductance of 80-nm-thick PTAA film before and after the O_2-light exposure for 1h.

Suppression of the degradation by light soaking in pure oxygen atmosphere

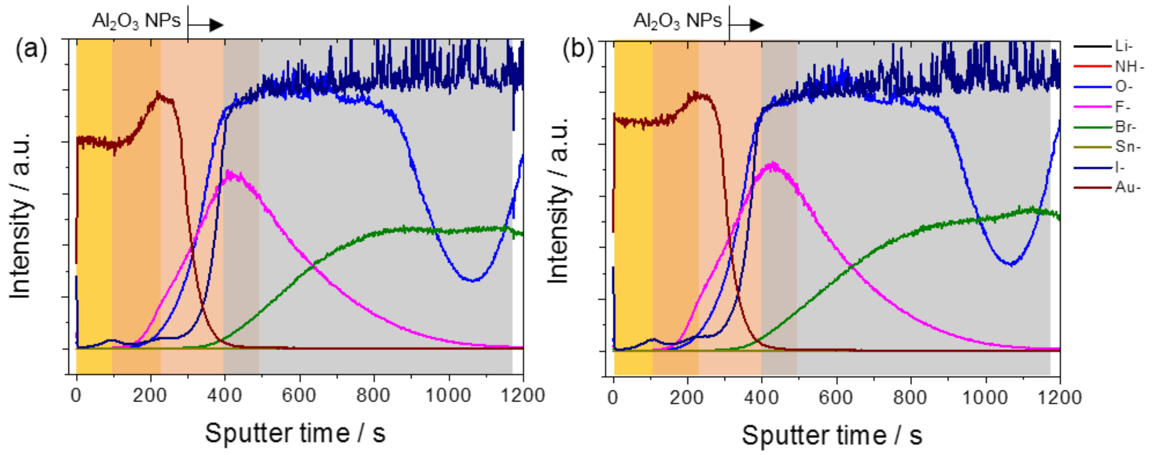

Figure S12. ToF-SIMS depth profiling of elements in PSCs with a *np*-Al_2O_3 layer between the perovskite and PTAA (a) before and (b) after 1 h of O_2-light exposure.

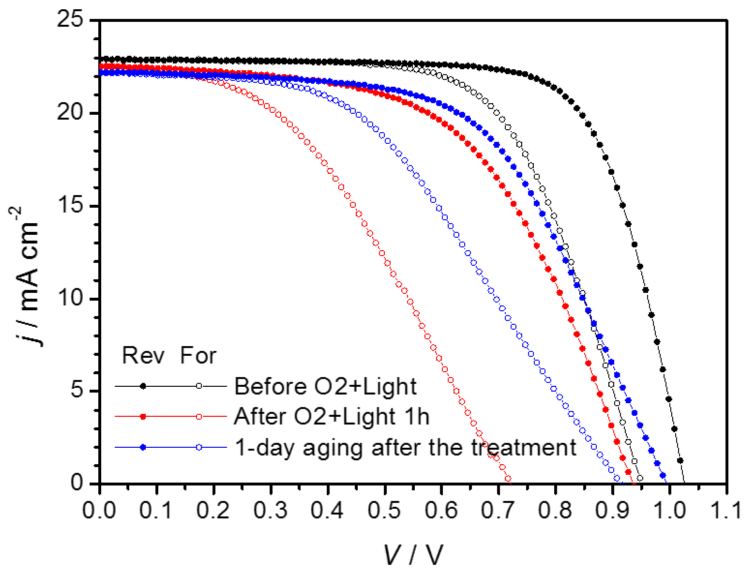

Figure S13. *j*-*V* curves before and after the O_2-light exposure for PSCs with the *np*-Al_2O_3 layer between PTAA and Au.
